# Early pathological characterization of murine dissecting abdominal aortic aneurysms

**DOI:** 10.1063/1.5053708

**Published:** 2018-12-20

**Authors:** Evan H. Phillips, Adam H. Lorch, Abigail C. Durkes, Craig J. Goergen

**Affiliations:** 1 Weldon School of Biomedical Engineering, Purdue University, West Lafayette, Indiana 47907, USA; 2 Department of Biology, Purdue University, West Lafayette, Indiana 47907, USA; 3 Department of Comparative Pathobiology, Purdue University, West Lafayette, Indiana 47907, USA; 4 Center for Cancer Research, Purdue University, West Lafayette, Indiana 47907, USA

## Abstract

We report here on the early pathology of a well-established murine model of dissecting abdominal aortic aneurysms (AAAs). Continuous infusion of angiotensin II (AngII) into apolipoprotein E-deficient mice induces the formation of aortic dissection and expansion at some point after implantation of miniosmotic pumps containing AngII. While this model has been studied extensively at a chronic stage, we investigated the early pathology of dissecting AAA formation at multiple scales. Using high-frequency ultrasound, we screened 12-week-old male mice daily for initial formation of these aneurysmal lesions between days 3 and 10 post-implantation. We euthanized animals on the day of diagnosis of a dissecting AAA or at day 10 if no aneurysmal lesion developed. Aortic expansion and reduced vessel wall strain occurred in animals regardless of whether a dissecting AAA developed by day 10. The aortas of mice that did not develop dissecting AAAs showed intermediate changes in morphology and biomechanical properties. RNA sequencing and gene expression analysis revealed multiple proinflammatory and matrix remodeling genes to be upregulated in the suprarenal aorta of AngII-infused mice as compared to saline-infused controls. Histology and immunohistochemistry confirmed that extracellular matrix remodeling and inflammatory cell infiltration, notably neutrophils and macrophages, occurred in AngII-infused mice with and without dissecting AAAs but not saline-infused controls. Understanding early disease processes is a critical step forward in translating experimental results in cardiovascular disease research. This work advances our understanding of this well-established murine model with applications for improving early diagnosis and therapy of acute aortic syndrome in humans.

## INTRODUCTION

Aortic rupture is an often fatal consequence of aortic dissections and aneurysms. The ultimate goal of aortic disease research is to prevent aortic rupture and decrease the high mortality associated with many types of aortic disease. Research efforts towards novel treatment strategies^1,2^ and biomarkers^3,4^ have been ongoing for many years, but there has been less emphasis on early versus later disease processes. Understanding early disease processes has the potential benefit of identifying high risk patients who would benefit from treatment of small aneurysms and do not meet the current criteria for surgical intervention.

Over the past 20 years, dissecting murine abdominal aortic aneurysms (AAAs) induced via angiotensin II (AngII)^5,6^ have become a valuable model for studying focal aortic dissection and expansion as well as intramural thrombus formation. These pathological features are often found in the thoracic aorta of humans with acute aortic syndrome.^7^ Conversely, human AAAs typically form below the level of the kidneys and exhibit slow expansion with gradual circumferential vessel wall destruction and remodeling.^8^ The AngII-induced dissecting AAA model also has features of atherosclerosis, inflammation, and extracellular matrix (ECM) remodeling, all of which are found in human aortic disease. While these have been well studied via histology and molecular analysis at a mature stage, it is difficult to study the early pathology of this model^9–11^ without knowing if and when an animal has already developed a focal dissection and aortic expansion. The aneurysmal lesions appear to develop suddenly on the order of minutes to hours,^12^ and therefore, determining the earliest time point in development is difficult. With increased access to small animal imaging systems, including micro-computed tomography,^13^ high-field magnetic resonance imaging,^14^ and high-frequency ultrasound (US),^15,16^ there have been multiple noninvasive longitudinal *in vivo* imaging studies examining experimental aortic disease models. However, AngII-induced dissecting AAAs show variable incidence rates,^17^ have abrupt and variable formation, and are not diagnosable without imaging prior to euthanasia. Some studies have thus misinterpreted AAA prevalence^18^ and possibly overlooked or misidentified aneurysmal lesions depending upon the criteria used.

Our previous imaging and characterization work with the AngII model motivated the present study. We previously tracked *in vivo* changes in aortic morphology and biomechanics in AngII-infused *apoE*^-/-^ mice over 28 days.^19^ While we measured progressive volumetric growth on average, we noted large differences, particularly in timing and volume, among animals. For this reason, we conducted a second study to investigate in more detail progression on an animal-specific basis.^20^ We first repeatedly screened the animals between days 3 and 21 post-implantation in order to determine the timing of initial aortic expansion for each mouse. Not all animals developed a dissecting AAA while others experienced sudden aortic rupture and died. We imaged each animal with a dissecting AAA for an additional 7 days after identification of initial aortic expansion. This study demonstrated that four lesions (identified on days 6 and 9 post-implantation) were heterogeneous in terms of early volumetric growth, thrombus deposition, false lumen flow, and hemodynamic metrics, including time averaged wall shear stress, oscillatory shear index, and formation of vortices.

For the present study, we implemented a daily US screening approach to diagnose aortic dissections within 24 h of formation. Thus, the physiological and molecular factors responsible for vessel wall remodeling are already active in these aneurysmal lesions. We hypothesize that similar factors in humans could be responsible for initiation of vessel wall remodeling and play a role in development. Furthermore, this work is of particular interest as the majority of patients diagnosed with AAAs have aortic diameters less than 5 cm and do not meet the criteria for surgical intervention. Indeed, understanding early AAA formation and progression could be helpful to improve translational strategies for the medical treatment of small aneurysms.

We compared differences in early disease progression among three experimental cohorts (AngII AAA, AngII No AAA, and saline) of male *apoE*^-/-^ mice at 12 weeks of age at baseline. Based on previous studies,^14,19–22^ we selected day 10 as the cutoff for screening AngII-infused animals that did not develop a dissecting AAA (AngII No AAA). We tested the hypothesis that a proinflammatory phenotype and microstructural defects develop in the suprarenal aorta as early as day 3 of AngII infusion and are sufficient to induce a dissecting AAA. We also hypothesized that the suprarenal wall would exhibit reduced circumferential cyclic strain (CCS) that is correlated with the dissection severity as seen by histology. Furthermore, based on a previous study by Usui *et al.*,^23^ we investigated whether animals in the AngII AAA cohort have higher aortic gene expression and activation of IL-1β, as compared to the AngII No AAA and saline cohorts. Finally, we investigated whether animals in the AngII No AAA cohort exhibit an intermediate state of inflammation and ECM remodeling that does not result in full vessel wall breakage. The results of this work provide a wealth of information on the early pathology of dissection and aortic expansion in the suprarenal mouse aorta. This study has implications for future research on acute aortic syndrome in humans and the investigation of potential biomarkers and treatment strategies for aortic disease.

## RESULTS

### AngII infusion led to abrupt dissecting AAA formation over a range of diagnosis days

Of the 15 AngII-infused animals, 7 developed a dissecting AAA by day 10 (47%; AngII AAA cohort), 5 did not develop a dissection (33%; AngII No AAA cohort), and 3 became moribund or experienced an aortic rupture (20%; excluded from study). None of the 5 saline-infused mice (Saline cohort) developed a dissecting AAA. Table I provides the breakdown of animal numbers and timing used to assign each animal. Two animals were diagnosed as early as day 3 post-implantation; none were diagnosed on either of the two final days (days 9 and 10). Classification to the AngII AAA or AngII No AAA cohort was made according to the daily check of B-mode, M-mode, and Color Doppler data (see the section on Methods). B-mode and M-mode data acquired at i) baseline, ii) on a screening day, iii) on a diagnosis day, and iv) on day 10 are provided for visual reference (supplementary material, Fig. 1 and Movie I).

**TABLE I.** Experimental design for *in vivo* monitoring and euthanasia. Twelve animals were assigned to either cohort 1 (AngII AAA) or 2 (AngII No AAA) depending on whether a dissecting AAA was identified by day 10 after AngII pump implantation. Three additional animals were monitored but excluded from the study. Two of these animals were moribund at day 5, and one experienced an aortic rupture on day 6 before a full US dataset was collected. Five animals in cohort 3 (saline) were implanted with saline-filled pumps. The number of animals (***d***) in cohort 1 that were diagnosed on a given day is bolded and italicized.

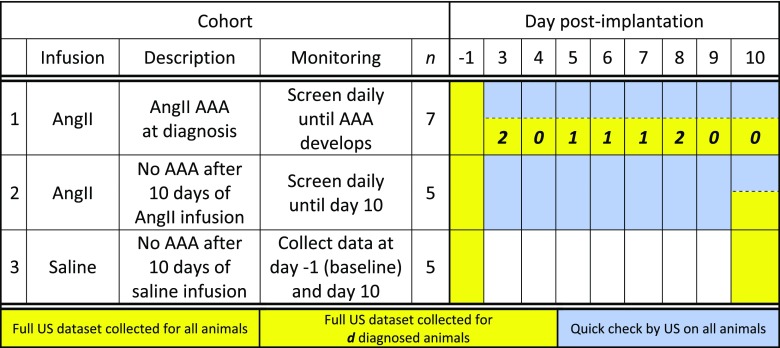

Systolic blood pressure (SBP) for AngII-infused animals increased modestly but significantly from baseline values after at least 6 days of infusion (supplementary material, Table I). Baseline values ranged from 86 to 108 mm Hg across all animals (AngII AAA: 97.4 ± 5.7 mm Hg; AngII No AAA: 101.8 ± 4.5 mm Hg; Saline: 95.6 ± 6.7 mm Hg). SBP increased to 124.5 ± 8.5 mm Hg (n = 8 of the 12 animals), and the averages were similar between the AngII AAA and AngII No AAA cohorts (118.7 ± 2.1 mm Hg and 128 ± 9.1 mm Hg, respectively). The change in SBP was minimal for the two animals measured in the Saline cohort.

### AngII infusion led to volumetric growth and strain reduction regardless of the dissecting AAA status

**FIG. 1.**
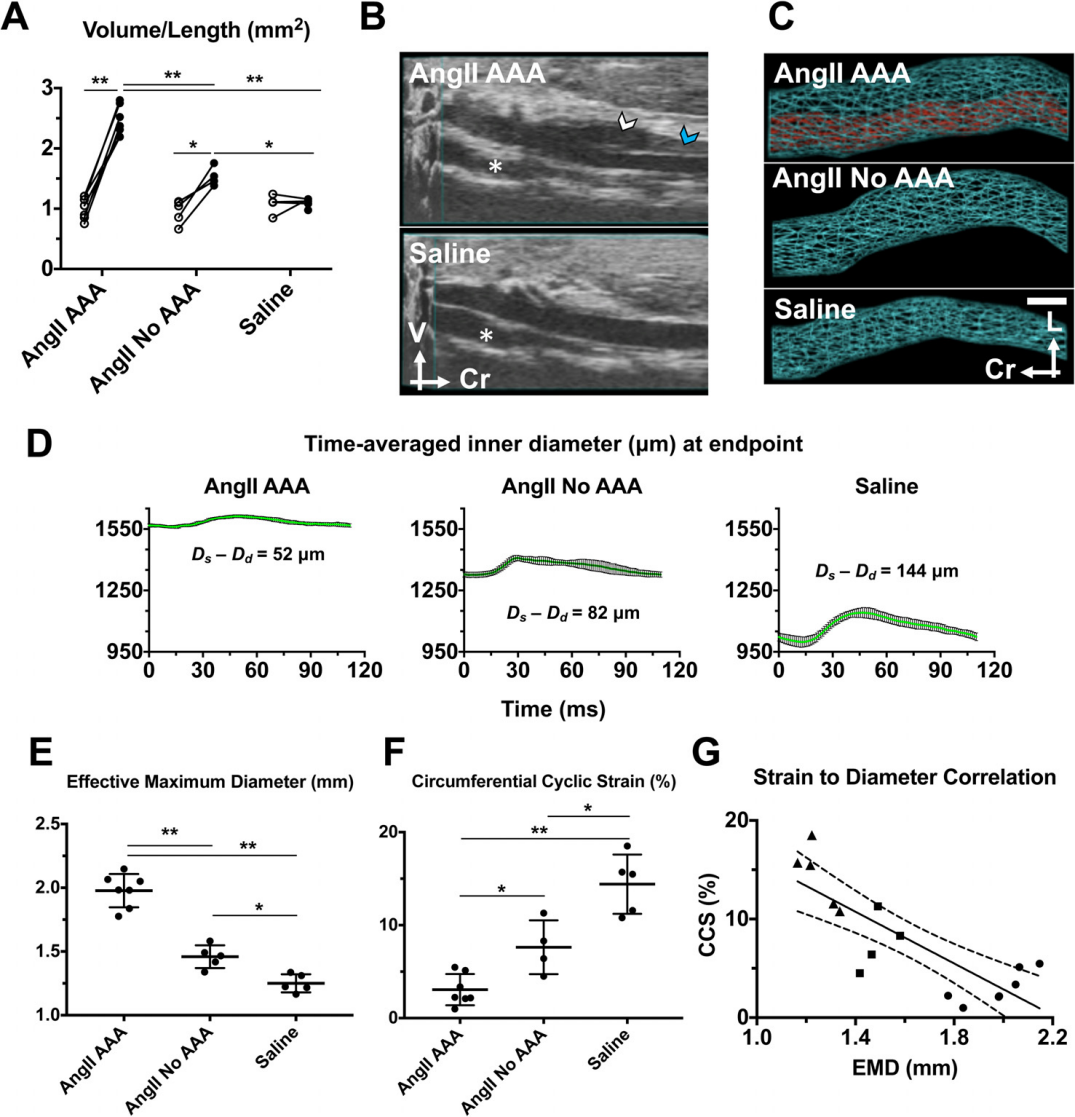
Volumetric growth and reduction in vessel wall strain occur with exogenous AngII infusion regardless of dissecting AAA status. (a) Individual volume/length values at baseline and the animal-specific endpoint across AngII AAA, AngII No AAA, and Saline cohorts. 2-way analysis of variance (ANOVA) with Sidak multiple comparisons. *p < 0.05; **p < 0.001. Open circles: baseline; Black circles: endpoint. (b) Example 3D B-mode data from the AngII AAA and Saline cohorts. White arrowhead: false lumen. Blue arrowhead: intramural thrombus. Asterisk: inferior vena cava; V: ventral; Cr: cranial. (c) Representative volumetric renderings reveal the complex morphology in a leftward-expanding lesion from an AngII AAA cohort mouse, vessel expansion without a focal dissection or thrombus from an AngII No AAA mouse, and a healthy-looking aorta from a Saline cohort mouse. In the AngII AAA example, the space between the true lumen (red) and the outer aortic wall (cyan) is the expanded media and adventitia resulting from a focal aortic dissection. Scale bar: 1 mm. L: left. Cr: cranial. (d) Representative time-averaged inner diameter measurements from each animal cohort. Each plot displays mean inner diameter values ± SD over the course of a cardiac cycle. The difference between the peak systolic (*D_s_*) and end diastolic (*D_d_*) inner diameters is shown for each example. (e) Effective maximum diameter (EMD) values at the animal-specific endpoint. One-way ANOVA with Tukey multiple comparisons. (f) Individual circumferential cyclic strain (CCS) values at the animal-specific endpoint. One-way ANOVA with Tukey multiple comparisons. (g) Correlation between vessel strain and the aortic diameter. CCS values at the animal-specific endpoint are plotted against the corresponding EMD values (triangles: Saline cohort; squares: AngII No AAA cohort; circles: AngII AAA cohort). Linear regression (solid line) and 95% confidence interval (dashed lines) are shown. Slope: −13.11. Pearson correlation coefficient (r): −0.82. *p* = 0.0001. *p < 0.05; **p < 0.001.

The average volume/length of the suprarenal aorta increased significantly from baseline for both the AngII AAA (140.9% ± 29.4%; *p *<* *0.001) and AngII No AAA (66.7% ± 16.4%, *p *<* *0.05) cohorts. Interestingly, there were statistically significant differences among all three cohorts at the animal-specific endpoint: AngII AAA and AngII No AAA (59.6% ± 8.7%; *p *<* *0.001); AngII AAA and saline (122.5% ± 15.2%; *p *<* *0.001); and AngII No AAA and saline (39.4% ± 5.0%; *p *<* *0.05) [Fig. 1(a)].

Volume/length values increased by more than 100% in six of the seven AngII AAA animals (108%–217%). Individual values for the combined false lumen and thrombus volume (i.e., total dissecting AAA volume minus true lumen volume) normalized by the length of the final dissecting AAA were similar across animals and constituted a large proportion of the total AAA volume (80% ± 6%; 73%–89%). For the seven dissecting AAAs, the axial lengths varied between 4.41 mm and greater than 14.92 mm (9.72 ± 3.18 mm) and the distal end of the aneurysmal lesions was observed both above and below the right renal artery. For one case, abdominal gas obscured the aorta proximal to the superior mesenteric artery at the endpoint and the volume was estimated in this region. Volumetric renderings calculated from 3D US segmentations [Fig. 1(b)] reveal the differences in vessel expansion among the three cohorts [Fig. 1(c)]. By comparison, upon postmortem inspection of the vessels *in situ*, the intermediate expansion and absence of a false lumen in the AngII No AAA cohort were not apparent.

We also investigated the differences in the diameter and vessel strain among the three cohorts. Based on time-averaged inner diameter values, we noted larger differences between peak systole and end diastole in the AngII No AAA and Saline cohorts as compared to the AngII AAA cohort [Fig. 1(d)]. In the examples shown, the difference between the peak systolic and end diastolic inner diameter was 52 *μ*m (AngII AAA), 82 *μ*m (AngII No AAA), and 144 *μ*m (Saline). As with the volume/length, the average effective maximum diameter (EMD) was significantly different among all three cohorts [Fig. 1(e)]. The average EMD was 35.5% ± 3.2% larger for the AngII AAA cohort relative to the AngII No AAA cohort (*p *<* *0.001). Relative to the Saline cohort, average EMD was 58.1% ± 5.1% larger for the AngII AAA cohort (*p *<* *0.001) and 16.7% ± 1.4% larger for the AngII No AAA cohort (*p *<* *0.05). The smallest EMD measurement for the AngII No AAA cohort (1.34 mm) was the same as the largest measurement for the Saline cohort, and not surprisingly, these two cohorts had the closest ranges of values. Average circumferential cyclic strain (CCS) values were significantly different among all three cohorts (AngII AAA: 3.07% ± 1.68%; AngII No AAA: 7.63% ± 2.89%; Saline: 14.42% ± 3.19%) [Fig. 1(f)]. For the AngII AAA cohort, average CCS was 78.7% ± 46.4% lower relative to the Saline cohort (*p *<* *0.001). For the AngII No AAA cohort, average CCS was reduced (−47.1% ± 20.7%; *p *<* *0.05) compared to the Saline cohort and was 59.8 ± 39.8% higher (*p *<* *0.05) than that for the AngII AAA cohort. The individual strain values for the AngII No AAA cohort fall in the range of values of 4.5%–11.3% which overlaps with values from the other two cohorts. Furthermore, we found CCS to be inversely correlated with EMD (linear regression with a slope of −13.11 and a Pearson correlation coefficient of −0.82). However, for larger diameter aortas, some strain values were outside of the 95% confidence interval [Fig. 1(g)].

### Inflammation- and extracellular matrix-related genes are differentially expressed among experimental cohorts

**FIG. 2.**
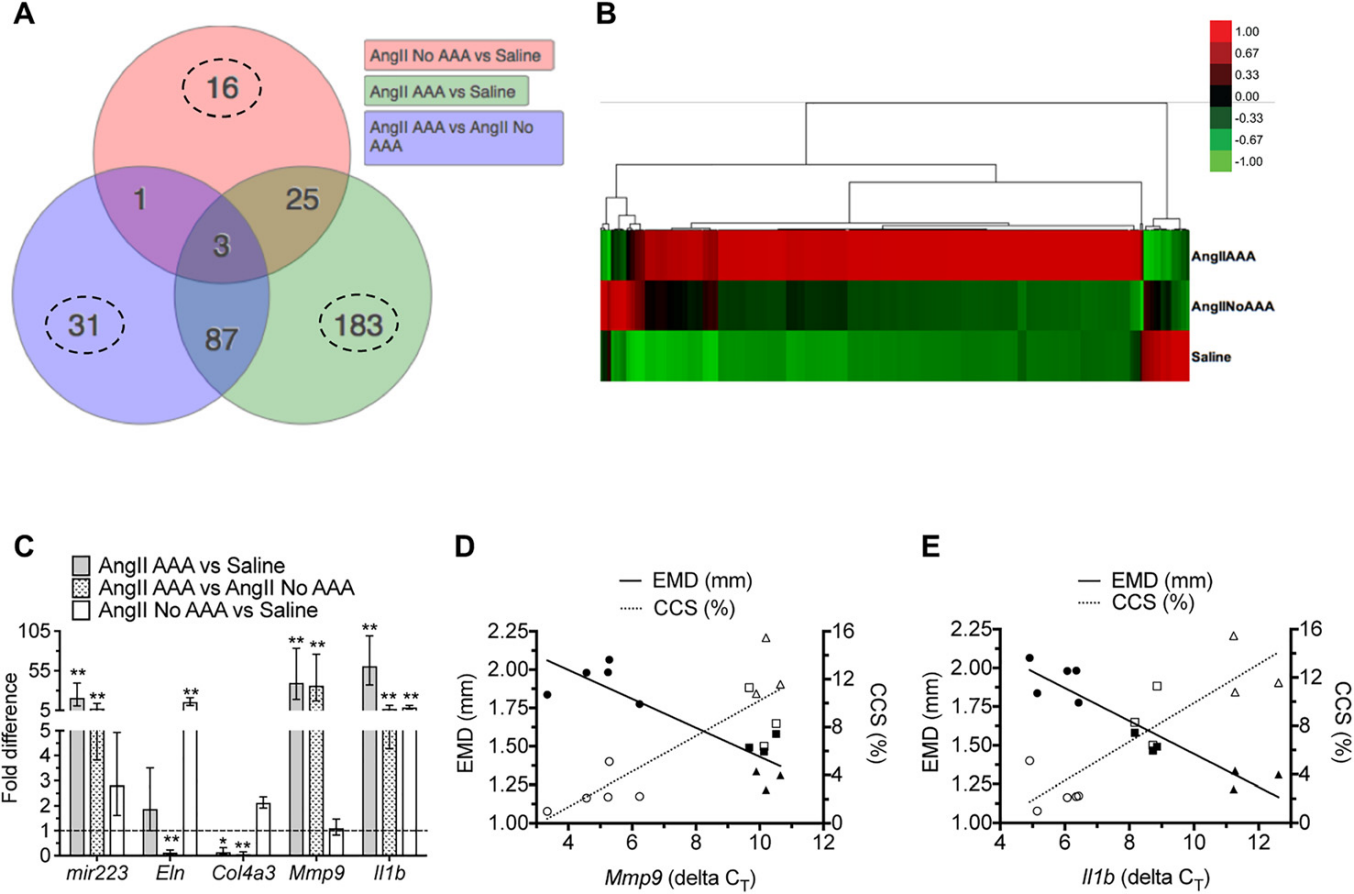
Gene expression analysis across cohorts. (a) Venn diagram of 346 significant differentially expressed genes (DEGs) selected according to a fold change greater than 1.5 and an adjusted p-value less than 0.1. The number of uniquely expressed genes (dashed line circles) is highest for the comparison with the highest expected contrast and lowest for the comparison with the lowest expected contrast. (b) Heat map and dendrogram of 346 DEGs across comparisons. Raw z-scores are color-coded. Scale bar: upregulated (red), downregulated (green), and no change (black). (c) Real-time PCR validation of selected DEGs (*mir223*, *Eln*, *Col4a3*, *Mmp9*, and *Il1b*) across comparisons. *Ppia* was first validated and used as a housekeeping gene for each run. Each assay was run in duplicate, and 3–5 biological replicates per cohort were used. One-way ANOVA with Holm-Sidak multiple comparisons of each cohort (*p < 0.05; **p < 0.001). (d) and (e) Linear regression analysis between the diameter, strain, and gene expression of *Mmp9* and *Il1b* across three cohorts. EMD (mm) and CCS (%) values are plotted against the corresponding animal-specific delta C_T_ values (housekeeping gene-corrected cycle threshold values for a target gene) for (d) *Mmp9* and (e) *Il1b*. Note that higher C_T_ values represent longer amplification and lower relative expression. Pearson correlation coefficients (r): −0.89 (*Mmp9* vs EMD); 0.86 (*Mmp9* vs CCS); −0.94 (*Il1b* vs EMD); 0.87 (*Il1b* vs CCS). Solid symbols: EMD values; open symbols: CCS values; circles: AngII AAA cohort; squares: AngII No AAA cohort; triangles: Saline cohort. Two data points from the AngII AAA cohort were excluded as outliers.

We found 346 differentially expressed genes (DEGs) to be significant for at least one of the experimental contrasts in the suprarenal aorta dataset. We applied two lines of evidence for DEG selection: (1) genes with a fold change of greater than 1.5 and (2) an adjusted *p*-value (false discovery rate) of less than 0.1. The highest number of unique DEGs (183) was found for the comparison with the highest expected contrast (AngII AAA vs. Saline) and the lowest number (16). for the comparison with the lowest expected contrast [AngII No AAA vs. saline; Fig. 2(a)]. These genes displayed three obvious patterns of expression across the experimental contrasts [Fig. 2(b)]. The first pattern (approximately 87% of DEGs) showed upregulated expression in the AngII AAA cohort, downregulated or no change in expression in the AngII No AAA cohort, and downregulated expression in the Saline cohort. The second pattern (8% of DEGs) showed downregulation for the AngII AAA cohort, little to no change in expression for the AngII No AAA cohort, and upregulation for the Saline cohort. Finally, the third pattern (5% of DEGs) showed little to no change in expression for the AngII AAA cohort, upregulation for the AngII No AAA cohort, and downregulation for the Saline cohort.

Of the three possible comparisons, we focused in particular on AngII AAA vs. AngII No AAA. Among the most upregulated genes (Table II) were primarily ones encoding for inflammatory factors and receptors (e.g., *Il1r2*, *Csf3r, Ccl2, Pf4*, and *Ccr1*) and matrix metalloproteinases (*Mmp8* and *Mmp9*). We also measured upregulation of an inflammation-related microRNA (*mir223*). Among the most downregulated genes (Table III) were ones encoding extracellular matrix proteins (e.g., *Optc*, *Col4a3, Eln, Mfap4*, and *Fmod*), cytoskeletal proteins (e.g., *Tnnt2* and *Cnn1)*, as well as both a peptidase inhibitor (*Serpina1d*) and a disintegrin and metalloproteinase with thrombospondin motifs (*Adamts8*). Not surprisingly then, several genes relevant to inflammation and extracellular matrix remodeling are differentially expressed in the AngII AAA cohort. A list of all DEGs with fold change values is provided in the supplementary material, Table II. We verified the differential expression levels of selected DEGs by real-time polymerase chain reaction (RT-PCR) [Fig. 2(c)]. These genes followed the same pattern of expression as seen with RNA-seq analysis. *mir223* was significantly upregulated in the AngII AAA cohort relative to each of the other cohorts, while *Il1b* was significantly upregulated in both the AngII and AngII No AAA cohorts. *Eln* was significantly downregulated for the AngII AAA cohort relative to the AngII No AAA cohort, but significantly upregulated for the AngII No AAA cohort relative to the Saline cohort. For the AngII AAA cohort, *Col4a3* had a significantly lower level of expression relative to each of the other cohorts, which was opposite to *Mmp9* expression levels.

**TABLE II.** Top 30 upregulated genes with the associated log_2_ fold change for AngII AAA vs. AngII No AAA comparison.

Top 30 upregulated genes (AngII AAA vs. AngII No AAA)		
Gene name	Full name	log_2_ (fold change)
Hsd3b1	Hydroxy-delta-5-steroid dehydrogenase, 3 beta- and steroid delta-isomerase 1	6.03
Cyp21a1	Cytochrome P450, family 21, subfamily a, polypeptide 1	5.23
Il1r2	Interleukin 1 receptor, type II	5.05
S100a8	S100 calcium binding protein A8 (calgranulin A)	4.94
S100a9	S100 calcium binding protein A9 (calgranulin B)	4.85
Arg1	Arginase 1	4.85
Cyp11a1	Cytochrome P450, family 11, subfamily a, polypeptide 1	4.77
Chil3	Chitinase-like 3	4.73
Ppbp	Pro-platelet basic protein;	4.73
	Chemokine (C-X-C motif) ligand 7	
Csf3r	Colony stimulating factor 3 receptor	4.60
Cyp11b1	Cytochrome P450, family 11, subfamily b, polypeptide 1	4.52
Mmp8	Matrix metalloproteinase 8	4.42
	Neutrophil collagenase	
Akr1b7	Aldo-keto reductase family 1, member B7	4.42
Star	Steroidogenic acute regulatory protein	4.33
Ccl2	Chemokine (C-C motif) ligand 2	4.21
	Monocyte chemoattractant protein 1	
Slfn4	Schlafen 4	4.16
Clec4d	C-type lectin domain family 4, member d	4.12
Pf4	Platelet factor 4	4.09
	Chemokine (C-X-C motif) ligand 4	
Hmox1	Heme oxygenase 1	4.07
Spp1	Secreted phosphoprotein 1	3.99
	Osteopontin	
Cxcl5	Chemokine (C-X-C motif) ligand 5	3.99
Mmp9	Matrix metalloproteinase 9	3.97
Cxcr2	Chemokine (C-X-C motif) receptor 2	3.93
	Interleukin 8 receptor, alpha	
F630028O10Rik,Mir223	RIKEN cDNA F630028O10 gene miR-223	3.88
Nrgn	Neurogranin	3.70
Pram1	PML-RAR alpha-regulated adaptor molecule 1	3.65
Clec4n	C-type lectin domain family 4, member n	3.64
	Dectin-2	
Ccr1	Chemokine (C-C motif) receptor 1	3.62
Cxcl2	Chemokine (C-X-C motif) ligand 2	3.61
Ifitm1	Interferon induced transmembrane protein 1	3.59

**TABLE III.** Top 30 downregulated genes with the associated log_2_ fold change for AngII AAA vs. AngII No AAA comparison.

Top 30 downregulated genes (AngII AAA vs AngII No AAA)		
Gene name	Full name	log_2_ (fold change)
Sost	Sclerostin	−3.98
Tnnt2	Troponin T2, cardiac	−3.97
1700003D09Rik	RIKEN cDNA 1700003D09 gene	−3.14
Optc	Opticin	−2.93
Pgbd1	piggyBac transposable element derived 1	−2.73
Serpina1d	Serine (or cysteine) peptidase inhibitor, clade A, member 1D	−2.69
Igfbp2	Insulin-like growth factor binding protein 2	−2.64
Casq1	Calsequestrin 1	−2.56
Lhx9	LIM homeobox protein 9	−2.49
Samd7	Sterile alpha motif domain containing 7	−2.45
Drd1	Dopamine receptor D1	−2.44
9330102E08Rik	RIKEN cDNA 9330102E08 gene	−2.38
Pnoc	Prepronociceptin	−2.29
Col4a3	Collagen, type IV, alpha 3	−2.27
Vax2os	Ventral anterior homeobox 2, opposite strand	−2.27
Cnn1	Calponin 1	−2.26
Eln	Elastin	−2.24
Mfap4	Microfibrillar-associated protein 4	−2.20
Rp1l1	Retinitis pigmentosa 1 homolog like 1	−2.14
Clec3b	C-type lectin domain family 3, member b	−2.12
Fmod	Fibromodulin	−2.11
Adamts8	A disintegrin-like and metallopeptidase (reprolysin type) with thrombospondin type 1 motif, 8	−2.10
Chmp4c	Charged multivesicular body protein 4C	−2.07
Trim71	Tripartite motif-containing 71	−2.06
Ccdc141	Coiled-coil domain containing 141	−2.05
Itgbl1	Integrin, beta-like 1	−2.05
Adamtsl2	ADAMTS-like 2	−2.03
Nov	CCN3 Nephroblastoma overexpressed gene	−1.98
Dusp27	Dual specificity phosphatase 27 (putative) Gm209	−1.96
Susd5	Sushi domain containing 5	−1.95

### A subset of DEGs related to inflammation and extracellular matrix remodeling shows differential expression in the suprarenal aorta

We focused the list of DEGs further by excluding genes with differential expression in the infrarenal aortas of the same animals. Forty-five of the 346 genes had no differential expression in the infrarenal aortas from the AngII AAA cohort (supplementary material, Table II, second tab). Of these genes, 8 were upregulated and 1 was downregulated relative to the AngII No AAA cohort. Genes that encode for a member of a lymphocyte differentiation antigen superfamily expressed on neutrophils (*Ly6g6d*), a plasma membrane efflux pump (*Abcb1b*), and a proteoglycan involved in protease storage in hematopoietic cells (*Srgn*) were all upregulated. *Il1rn*, which encodes for the IL-1 receptor antagonist, was also upregulated. *Sost*, a gene that encodes for a Wnt/β-catenin pathway inhibitor, was downregulated.

Relative to the Saline cohort, 34 genes associated with multiple processes were upregulated, including inflammation (e.g., *Pf4, Mpeg1*, and *Tnfrsf1b*), reactive oxygen species production (*Cybb*), blood pressure regulation (*Ednrb*), thrombus formation (*F13a1* and *Emilin2*), extracellular matrix remodeling (e.g., *Mmp14*, *Adam12*, and *Col8a1*), and leukocyte recruitment (*Gcnt1*). *Lrg1*, a gene encoding a member of the leucine-rich repeat family of proteins, was upregulated and is a potential pathological biomarker. Individual genes associated with second messenger production (*Adcy5*), connective tissue assembly (*Thsd4*), immune homeostasis (*Cytl1*), and smooth muscle cell function (*Synpo2*) were downregulated.

Ten of the 346 genes had no differential expression in the infrarenal aortas from the AngII No AAA cohort. Eight genes were upregulated relative to the Saline cohort, such as *Sst*, which encodes for a hormone with immunosuppressive and antiproliferative effects. Other genes were not well annotated or have no supporting evidence to date for aortic expression. Relative to the AngII AAA cohort, *Ly6g6d* and *Hsd3b1* were downregulated.

### Suprarenal aorta Il1b expression is associated with the aortic size and strain

We analyzed the association between aortic gene expression and US-derived metrics of size (EMD and volume/length) and strain (CCS). As these metrics are related to the vessel structure and pulsatility, we first evaluated the relationship to expression levels of *Mmp9*, which can cleave elastin, collagen, and other proteins [Fig. 2(d)]. For 5 of the 7 AngII AAA animals, lower ΔC_T_ values (i.e., higher relative expression) of *Mmp9* were associated with larger EMD and lower CCS. For AngII No AAA and Saline animals, higher ΔC_T_ values were associated with lower EMD and higher CCS. Overall, regression lines showed good correlation (Pearson correlation coefficients of −0.89, −0.94, and 0.86 for EMD, Volume/Length, and CCS, respectively). *Mmp9* expression levels, however, overlapped for the AngII No AAA and Saline cohorts.

We then evaluated the relationship to *Il1b*, which had a wider range of expression values [Fig. 2(e)]. Intermediate ΔC_T_ values of *Il1b* for the AngII No AAA animals were also associated with moderate vessel expansion and strain reduction. Regression lines again showed good correlation (Pearson correlation coefficients: −0.94, −0.92, and 0.87).

### Regulation of neutrophil migration, neutrophil chemotaxis, monocyte chemotaxis, blood coagulation, and cellular extravasation are enriched in dissecting AAAs

The Database for Annotation, Visualization and Integrated Discovery (DAVID) system calculated high enrichment scores for the AngII AAA cohort in functional annotation clusters related to the immune system, chemokines, and secreted and extracellular factors. Relative to the Saline cohort, high enrichment scores for the AngII No AAA cohort included functional annotation clusters related to secreted and extracellular factors, the extracellular matrix, immune system processes, and collagen.

**FIG. 3.**
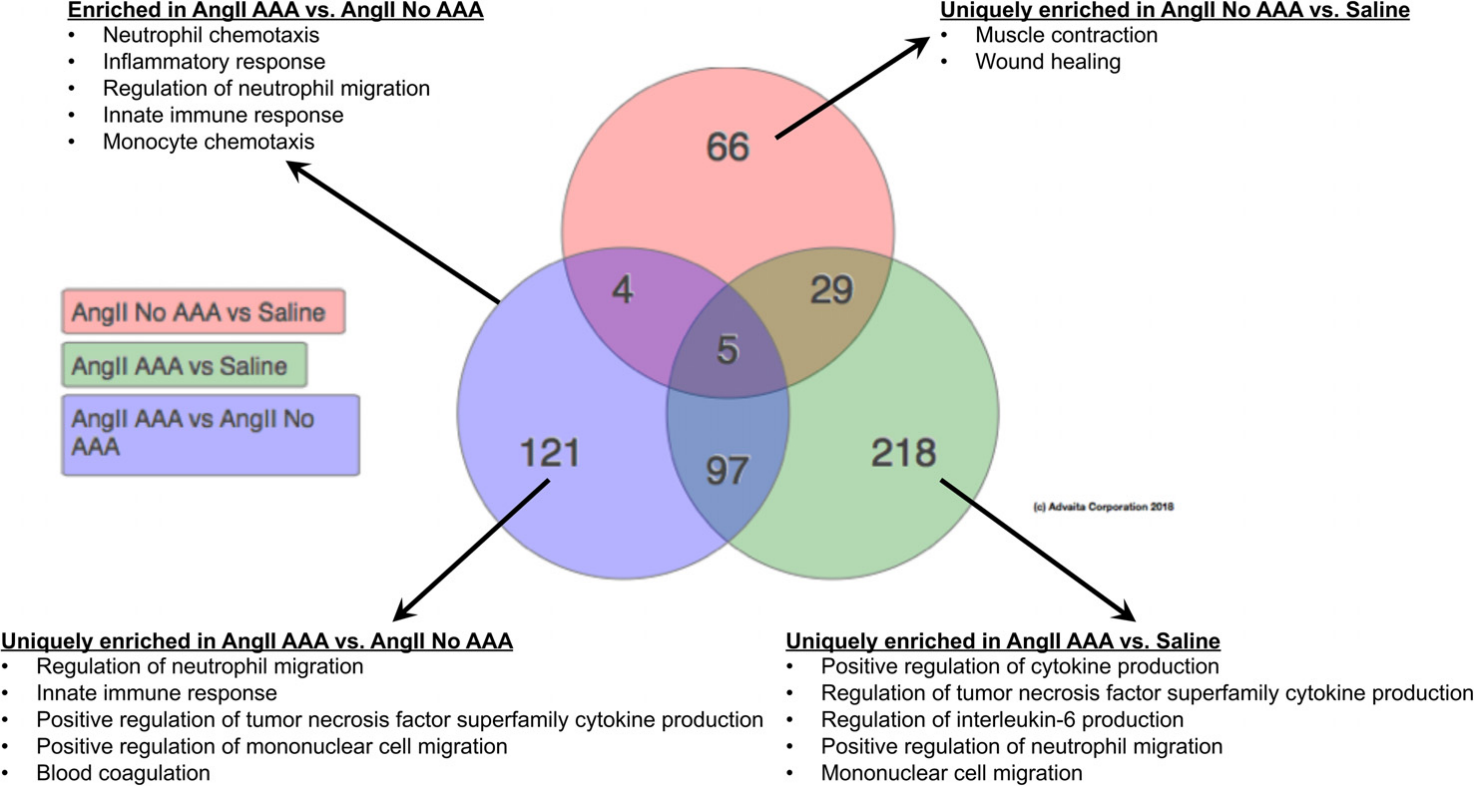
Enriched gene ontology (GO) terms for biological processes across cohorts. Venn diagram showing the numbers of unique and common GO terms for biological processes across cohorts. Example biological processes from various comparisons are displayed. Adjusted *p* < 0.05 (using weight pruning correction) was used as a threshold for statistical significance.

Statistical ranking of gene ontology (GO) terms for biological processes revealed that multiple inflammatory processes were highly enriched in the AngII AAA cohort compared to both the AngII No AAA and Saline cohorts (Fig. 3). Interestingly, multiple GO terms identified regulation of neutrophils as a significant biological process. Furthermore, GO terms for cytokine production, mononuclear cell migration, and monocyte chemotaxis were enriched, supporting the expected presence and recruitment of monocytes/macrophages. In line with US findings and necropsy images of thrombus in all dissecting AAAs, processes for blood coagulation were also enriched. Collagen metabolism and cellular extravasation were uniquely enriched in the AngII AAA cohort as compared to the AngII No AAA cohort. The AngII No AAA cohort was uniquely enriched for processes including muscle contraction and wound healing. A complete list of all significant GO terms for biological processes as well as molecular functions across the three comparisons is provided in the supplementary material, Table III. GO term analysis with the subset of 45 suprarenal-specific AngII AAA DEGs produced a shorter list of biological processes that included regulation of Rho-dependent protein serine/threonine kinase activity (GO:2000298), positive regulation of tumor necrosis factor production (GO:0032760), inflammatory response (GO:0006954), and immune response (GO:0006955).

### Comparative analysis of differentially expressed genes after acute and chronic AngII infusion

We compared DEGs in the present study with those identified in a chronic (28-day) AngII infusion study (GEO accession # GSE12591).^24^ Forty-four DEGs were found to be unique to the present study, including factors involved in inflammation (e.g., *Ccl6*, *Ccr2*, and *Arg1*), reactive oxygen species generation (e.g., *Ncf1* and *Lyz2*), and regulation of transcription (*Spi1*) (supplementary material, Table IV and Fig. 2). AngII has been shown to stimulate oxidative stress, which is proposed to be a driver for dissecting AAA development.^23,25^ We measured upregulated expression of the nicotinamide adenine dinucleotide phosphate (NAPHD) oxidase subunit p47phox (*Ncf1*)^26^ and lysozyme M (*Lyz2*)^27^ in the AngII AAA cohort. Other DEGs from this list have yet to be studied in the context of aortic disease but are relevant to neutrophil biology. Serglycin (*Srgn*) is a proteoglycan required for granule storage of neutrophil elastase,^28^ and PRAM-1 (*Pram1*) is an adaptor molecule expressed mostly in mouse neutrophils which is necessary for reactive oxygen species production.^29^ Several DEGs found from our comparative analysis to the chronic AngII infusion study therefore include potential early therapeutic targets.

### Histology and immunohistochemistry

**FIG. 4.**
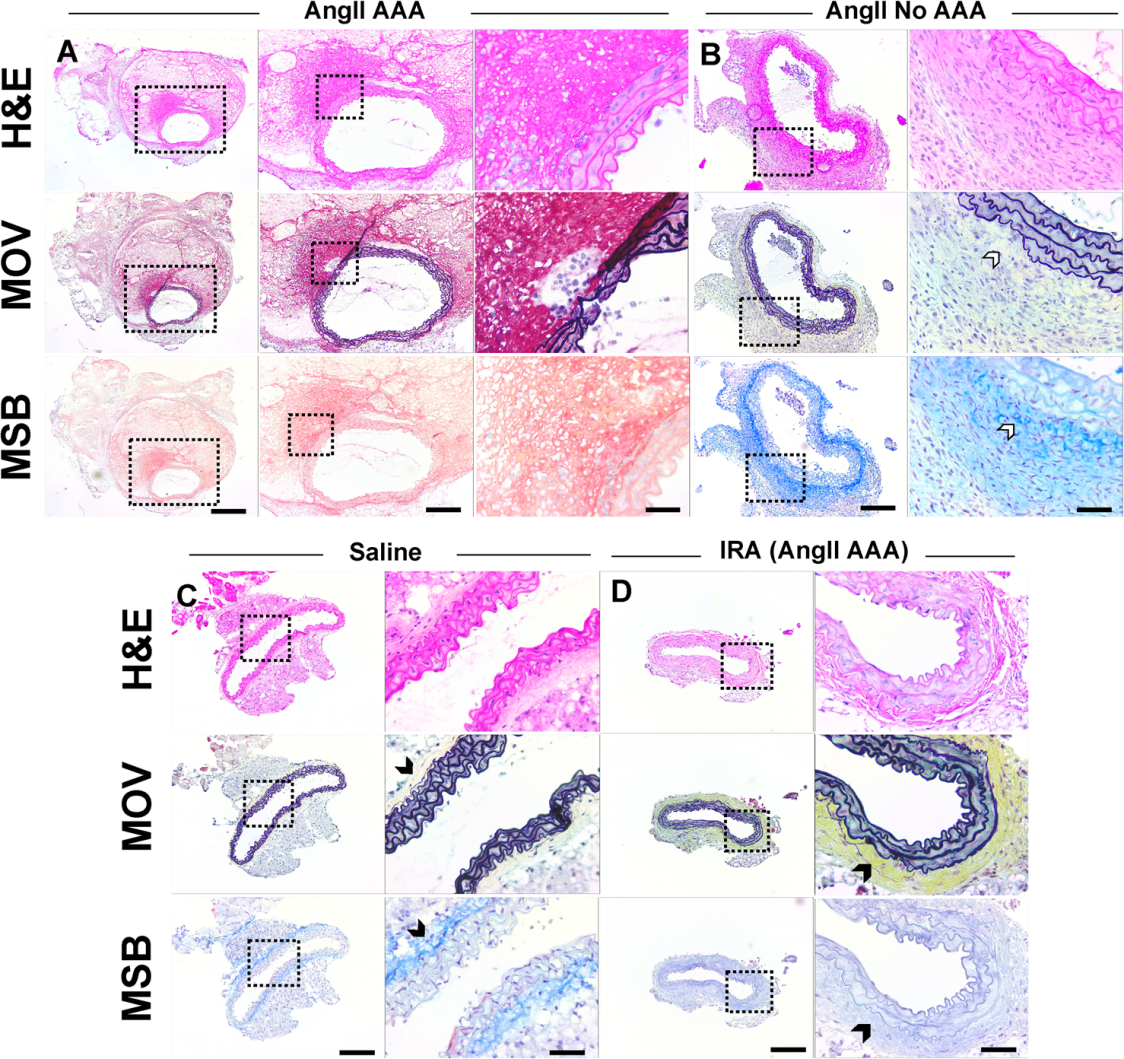
Hematoxylin and Eosin (H&E), Movat's Pentachrome (MOV), and Martius, Scarlet and Blue (MSB) aortic tissue histology. (a) MOV staining of a dissecting AAA (AngII AAA cohort) shows focal breakage of elastin (black). MSB staining shows an intramural thrombus by positive fibrin (red) and red blood cell (yellow) stains in proximity to the focal dissection identified. Very minimal adventitial collagen can be seen in this sample. Suprarenal aortas from the AngII No AAA (b) and Saline (c) cohorts show only minor microstructural defects in elastin lamellar units. Minimal adventitial collagen (white arrowheads) is observed for vessels from the AngII No AAA cohort, while the Saline cohort (c) and some infrarenal aortas (IRA) from the AngII AAA cohort (d) have more adventitial collagen (black arrowheads). AngII AAA scale bars: 500, 200, and 50 *μ*m. AngII No AAA, Saline, and IRA (AngII AAA) scale bars: 200 and 50 *μ*m.

Hematoxylin and eosin (H&E), Movat's Pentachrome (MOV), and Martius, Scarlet and Blue (MSB) staining revealed focal elastin breakage, a moderate number of inflammatory cells, and fibrin in the suprarenal aortas of the AngII AAA cohort (Fig. 4). For one mouse, we confirmed that a dissecting AAA extended distal to the renal arteries. Suprarenal aortas from the AngII No AAA cohort did not have evidence of aortic dissection or aneurysm formation with the possible exception of one tissue showing separation of the adventitia from media and the presence of eosinophilic fibrillar material. However, this could be due to a tissue processing artifact as no dissection was observed by US or after euthanasia. Interestingly, tissues from the AngII AAA and AngII No AAA cohorts exhibited minimal adventitial collagen as compared to the Saline cohort (MOV and MSB in Fig. 4). The infrarenal aortas from the AngII AAA cohort had abundant adventitial collagen in three of the seven tissues.

**FIG. 5.**
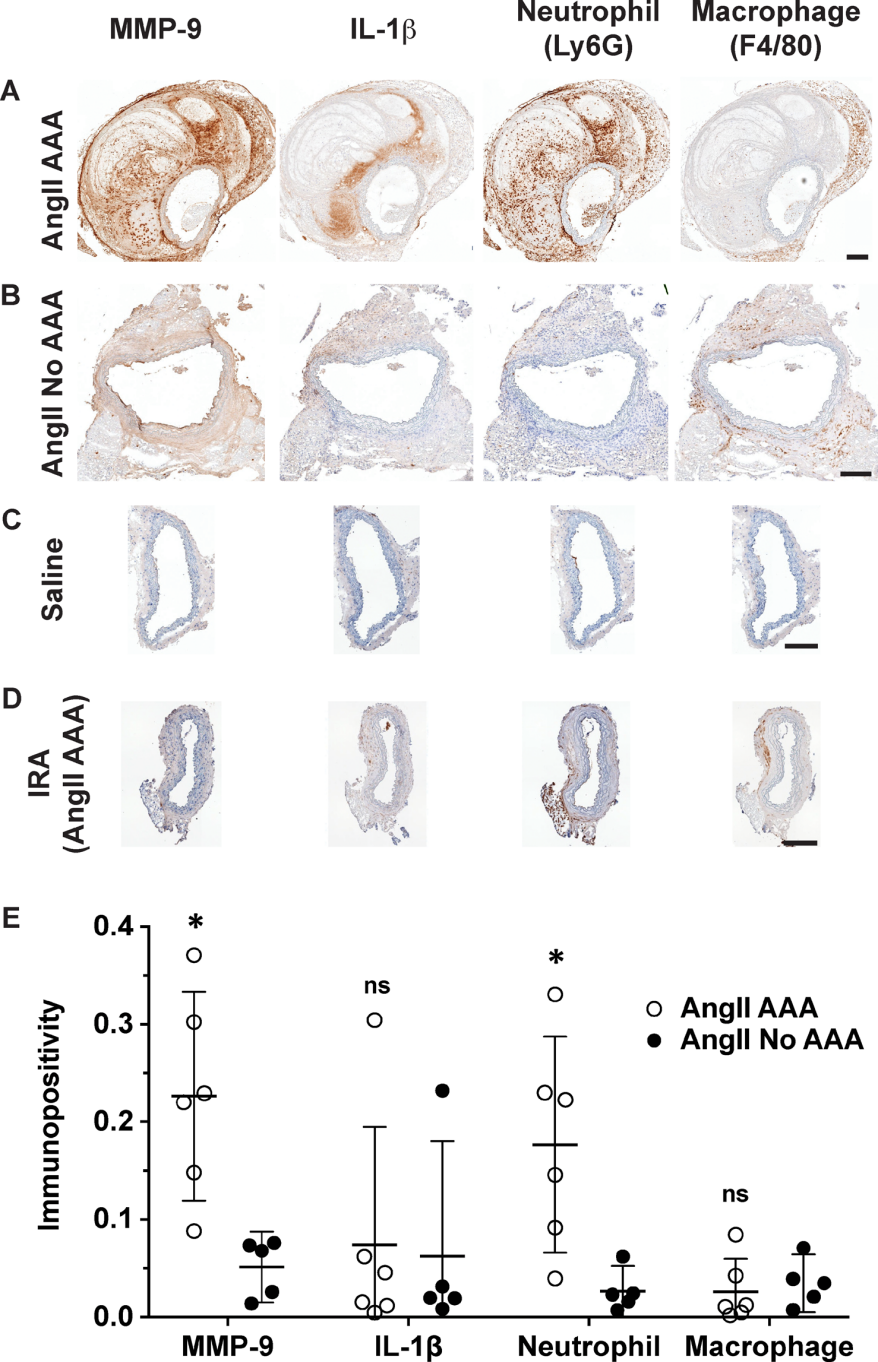
Immunohistochemistry (IHC) and semi-quantitation of MMP-9, IL-1β, neutrophils, and macrophages. (a)–(d) DAB single stain IHC of MMP-9, IL-1β, Ly6G^+^ neutrophils, and total macrophages (F4/80^+^) on example AngII AAA (a), AngII No AAA (b), Saline (c), and IRA (d) tissues. MMP-9 is co-distributed with neutrophils and macrophages in the adventitia (a). In the dissected region, neutrophils are abundant and MMP-9 and IL-1β are observed along the interface with the elastic lamina (a). Vessels from the Saline cohort (c) and the infrarenal aortas (IRA) from the AngII AAA cohort (d) show weak immunolabeling other than background and periadventitial staining. Scale bars: 200 *μ*m. (e) Semi-quantitation was performed on medium and strong immunopositive areas within the vessel wall. Unpaired t-tests were run to compare the AngII AAA and AngII No AAA cohorts (* *p* < 0.05; ns, not significant).

MMP-9 was abundant throughout the vessel wall for the AngII AAA cohort. Relative to the AngII No AAA cohort, MMP-9 percent immunolabeling was 5-fold greater on average (*p *=* *0.0071; Fig. 5). MMP-9 in the infrarenal aortas from the AngII AAA cohort was weak and non-specific, and for the Saline cohort, it had low intensity. IL-1β was less abundant in general and more sparsely distributed. For the AngII AAA cohort, IL-1β immunolabeling was variable with five of the seven dissecting AAAs having intense labeling that tapers away from the wall into the dissected region. The other two tissues had minimal labeling at all; however, in all seven tissues, we observed MMP-9 and IL-1β along the media/dissected region interface. For the AngII No AAA cohort, staining intensity and abundance of IL-1β were low except for the tissue with an apparent aneurysm where all the positive staining resided. IL-1β immunolabeling had low intensity and abundance and was largely confined to the adventitia for the Saline cohort and the infrarenal aortas from the AngII AAA cohort.

The adventitia of the aortas in the AngII AAA and AngII No AAA cohorts contained moderate numbers of mixed inflammatory cells, most notably neutrophils and macrophages (H&E in Fig. 4; Ly6G and F4/80 in Fig. 5), as well as lymphocytes and few plasma cells. For neutrophils in the AngII AAA cohort, we quantified 6-fold greater percent immunolabeling on average (*p *=* *0.017) throughout the vessel wall including the dissected region (Fig. 5). Macrophages had similar percent immunolabeling in both cohorts. The adventitia of the aortas from the Saline cohort contained inflammatory cells within normal limits. The inflammatory cells in the adventitia of the infrarenal aortas from the AngII AAA cohort were within normal limits except for the sample with a distally extending aneurysm and a tissue with mildly increased levels.

## DISCUSSION

We investigated the early pathology of dissecting AAAs by differentiating the morphological, biomechanical, microstructural, and inflammatory changes occurring in mice with and without suprarenal aortic dissection. We found that AngII exposure led to aortic vessel expansion and a reduction in vessel wall CCS. Neutrophil and mononuclear cell infiltration, IL-1β expression, and medial and adventitial ECM remodeling occurred to varying degrees dependent on whether an aortic dissection was identified *in vivo*. Below, we further discuss tissue- and gene-level differences identified in the AngII AAA and AngII No AAA cohorts.

### Aortic expansion and vessel wall pulsatility in the AngII No AAA cohort

Aortic vessel expansion and reduction in CCS occurred in all AngII-infused animals regardless of whether a dissecting AAA formed. This result was surprising for the AngII No AAA cohort because significant changes in the EMD or volume/length were not expected in the absence of a dissection. We attribute the increase in the EMD (42% ± 4%) and volume/length (67% ± 16%) in this cohort to the hypertensive effect of AngII on aortic remodeling.^30^ The percent increase in EMD was below a 50% threshold, the current clinical metric used to define human AAAs, for four of the five AngII No AAA murine aortas. Geometric measurements from the *in vivo* US data suggest that there is moderate vessel enlargement, especially after 10 days of AngII infusion. Despite the uniform lengths used for volumetric segmentation in this cohort, the percent increase for volume/length values ranged between 30% and 109%.

Endpoint CCS was less than 10% for three of the five animals in the AngII No AAA cohort (4.5%, 6.4%, and 8.3%) and overlapped with animals in the other two cohorts. Future work could include acquisition of 4D US data to analyze global and region-specific vessel wall strain in these aortas.^31^ This more sophisticated analysis would provide insight into where along the suprarenal aorta of AngII-infused animals we detect the largest reductions in pulsatility. During daily screening of AngII-infused animals, we collected M-mode data to assess whether average CCS was reduced. We performed measurements at three different axial locations along the suprarenal aorta but did not identify abnormally low CCS in any animals prior to the development of dissecting AAAs. Using 4D image analysis for this purpose however may have better predictive capability by correlating regional strain heterogeneity with potential for focal medial elastin breakage, dissection, and aortic expansion.

### Aortic gene expression of matrix remodeling enzymes and extracellular matrix proteins

We found high levels of suprarenal MMP expression, including *Mmp8* and *Mmp9*, in the AngII AAA cohort. MMP-8, expressed in neutrophils as well as endothelial cells, vascular smooth muscle cells, and macrophages, cleaves native type I and type III fibrillar collagens.^32–34^ *Mmp8* expression could be indicative of the presence of infiltrating neutrophils^35^ or synthesis by adventitial mesenchymal cells.^36^ Wilson *et al.* have shown that MMP-8 activity is significantly higher in areas where aortic rupture has occurred in human AAAs,^37^ supporting the theory that its specificity towards proteolysis-resistant fibrillar collagens is necessary for excess collagen breakdown and eventual rupture.^38^

There is clearly a significant role of MMP-9 in aortic disease initiation. Based on the finding of high circulating levels of MMP-9 in patients with acute aortic dissection, Kurihara *et al.* determined that AngII induced the release of MMP-9 from neutrophils, which led to acute aortic dissection in the thoracic aorta of mice that were administered a lysyl oxidase inhibitor.^39^ We verified in the present study that the highest intensity and abundance of MMP-9 were found in AngII AAA tissues (Fig. 5). Strong adventitial staining is observed and is codistributed with both F4/80^+^ macrophages and Ly6G^+^ neutrophils. Future work could include colocalization analysis of these markers and investigation of whether MMP-9, beyond its role in elastin degradation,^40^ is sufficient to activate IL-1β during initial aortic expansion.^41^

Interestingly, *Eln* expression was highest in the AngII No AAA cohort. We also measured differential expression of elastin-associated ECM proteins, such as *Fbn1* and *Mfap5*, suggesting ongoing vessel wall remodeling or repair processes involving elastin microfibril assembly.^42^ For AngII No AAA tissues, we observed intermittent and small microstructural defects to the elastin. One tissue did have two small transmural ruptures on the proximal end of the suprarenal aorta. While further work is needed, this initial analysis suggests that increased *Eln* expression may be protective against AngII-induced dissections.

Various collagen types showed differential expression and were classified based on related functions to other ECM and secreted factors. For the AngII AAA cohort, *Col8a1* was significantly upregulated relative to the Saline cohort and was functionally classified (i.e., shared multiple annotation terms in common) with genes encoding for complement pathway factors *C1q*^43^ and *C1qtnf6*^44^ as well as *Emilin2* (elastin microfibril interfacer 2).^45^ Increased local synthesis of EMILIN2 may be occurring in dissecting AAA formation; its proposed role in thrombus formation and contraction would be of interest to explore.^45^ We also noted downregulation of *Fmod*, which encodes a fibrosis-promoting collagen-associated protein, fibromodulin. Dense collagen formation is increased by fibromodulin, which promotes collagen cross-linking activity.^46^ This finding agrees with our histology which revealed only a thin layer of adventitial collagen in the AngII AAA tissues. Indeed, compensatory collagen deposition and remodeling may not be present at this early stage (cf. to Refs. 19 and 20).

### Inflammatory gene expression signatures

GO terms for inflammatory cell recruitment and regulation predominated for the AngII AAA cohort. Inflammation-resolving processes (e.g., wound healing and negative regulation of cytokine production) were identified for both the AngII AAA and AngII No AAA cohorts. We measured high expression levels of *Il1b* in AngII-infused animals with the largest difference for the AngII AAA cohort relative to the Saline cohort (∼60-fold increase; *p *<* *0.001). *Nlrp3*, which encodes for a sensor protein that is part of the inflammasome complex, was significantly upregulated relative to the AngII No AAA cohort as was the signaling receptor for IL-1β (*Il1r1*) relative to the Saline cohort. Relative to the AngII No AAA cohort, however, higher expression of some factors involved in blunting IL-1-mediated inflammation (*Il1r2* and *Il1rn*) suggests that a more complex picture is at a play within this early stage.

Martin *et al.* demonstrated that IL-1R2, a decoy signaling receptor for IL-1, is constitutively expressed on mouse neutrophils, and after an inflammatory stimulus, it can be shed from the membrane and then re-expressed.^47^ Thus, a soluble form of this receptor is able to sequester free IL-1. IL-1 receptor antagonist, encoded by *Il1rn* (IL-1Ra), is required in excess of IL-1β in order to inhibit IL-1-induced processes and is secreted by the same cells producing IL-1β.^48,49^ *Il1rn* was found to be a DEG in the AngII AAA suprarenal dataset that was absent in the infrarenal dataset, suggesting that a counterbalancing inflammatory response may be active in the suprarenal aorta. Ingenuity pathway analysis (IPA) canonical pathway analysis for the AngII AAA vs. Saline comparison identified the “inflammasome pathway” as activated (z-score 2.236; *p *=* *0.0035) similar to what we calculated using the publicly available results from the chronic (28-day) AngII infusion study^24^ (z-score 2; *p *=* *0.0164). “IL-1 signaling” had borderline activation (z-score 1.897; *p *=* *0.0012) based on differential expression of *Il1r1*, *Myd88*, *Fos*, *Ikbke*, *Irak3*, *Nfkbia*, *Gnai3*, and *Adcy5* and is more significant as compared to the chronic study (z-score 1.342; *p *=* *0.321). IL-1β immunolabeling had similar overall abundance in the AngII AAA and AngII No AAA cohorts; however, higher intensity in particular along the edge of the dissected regions was seen in the dissecting AAAs (Fig. 5). Taken together, these data suggest that the AngII AAA cohort shows upregulated expression of factors involved in the inflammasome pathway and IL-1β signaling and that IL-1β inhibition or sequestration may also be active.

Neutrophils and macrophages are the inflammatory cell types we found to show high and consistent enrichment in the AngII AAA cohort. A neutrophil marker (*Ly6g6d*) was a DEG in the suprarenal dataset that was absent in the infrarenal dataset. Other granulocyte markers (e.g., *Csf3r*) and factors promoting neutrophil chemotaxis (e.g., *Pf4* and *Cxcl2*)^50,51^ were also differentially expressed relative to the other two cohorts. IPA identified “fMLP signaling in neutrophils” (12 genes; z-score 3.464; *p *=* *2.36E-4) and “infiltration of neutrophils” (45 genes; z-score 3.015; *p *=* *1.2E-25) as activated for the AngII AAA vs. AngII No AAA comparison. These processes had lower activation z-scores for the chronic study (z-scores 1.89 and 1.358, respectively). Activation of N-formylmethionine leucyl-phenylalanine (fMLP) signaling in neutrophils raises the possibility that mitochondrial chemotactic peptides released upon cell injury and necrosis^52^ are responsible for neutrophil recruitment in the suprarenal aorta and subsequent pro-inflammatory or anti-inflammatory effects.^53^ Ly6G^+^ neutrophils were abundant throughout the adventitia and dissected region of AngII AAA tissues, similar to the distribution for the AngII No AAA tissues but which had less positive immunolabeling (Fig. 5). We also noted pervasive immunolabeling in the periadventitial space, which was excluded from analysis. Thus, gene- and tissue-level results indicate that neutrophil recruitment and infiltration are particularly active in the suprarenal aorta following acute AngII infusion. Laroumanie *et al.* found similar results after acute AngII infusion in a non-hyperlipidemic knockout model.^54^ For the present study, whether neutrophils are involved in dissection formation and/or subsequent vessel remodeling within the dissecting AAA is not clear.

Key chemokines and chemokine receptors on monocytes/macrophages showed high expression (e.g., *Ccl2*, *Ccr1*, and *Ccr2*) in the AngII AAA cohort. “Production of nitric oxide and reactive oxygen species in macrophages” (z-score 2.985; *p *=* *4.28E-10), “Fcγ receptor-mediated phagocytosis in macrophages and monocytes” (z-score 4.082; *p *=* *1.25E-16), and “CCR5 signaling in macrophages” (z-score 2.236; *p *=* *0.0202) were all found to be significantly activated for the AngII AAA vs. AngII No AAA comparison in the suprarenal dataset. For the infrarenal dataset, only “Fcγ receptor-mediated phagocytosis in macrophages and monocytes” was found to have borderline activation (z-score 2; *p *=* *0.0571). These three pathways showed very similar activation z-scores (3.051, 3.606, and 2.236, respectively) for the chronic infusion study. Total (F4/80^+^) macrophage immunolabeling on AngII AAA tissues was abundant throughout the adventitia and sparsely distributed in the dissected region (Fig. 5). These results confirm previous studies^12,21,27,55^ that macrophages are an active inflammatory cell type in the suprarenal aorta and underscore their involvement in early dissecting AAA development.

### Potential disease regulator biomarkers and therapeutic targets

Among the highest expressed DEGs in the AngII AAA cohort was *mir223* (∼7-fold average increase relative to the AngII No AAA cohort; *p *=* *0.0023), a micro-RNA which is released possibly exclusively by hematopoietic cells.^56^ This micro-RNA is a potential biomarker and therapeutic target, requiring further investigation in light of recent publications on multiple inflammatory diseases, including chronic obstructive pulmonary disease^57^ and cancer.^58^ Chu *et al.* recently proposed a mechanism for how miR-223 may be released from leukocytes and platelets into circulation and lead to vascular injury that causes Kawasaki Disease, a genetic condition associated with increased systemic vascular inflammation that can cause coronary artery aneurysms.^56^ Kin *et al.* have shown that relative to healthy patients, higher *mir223* expression is detectable in human AAA tissue and accompanied by lower expression in AAA patients' plasma.^59^

The most downregulated DEG in the AngII AAA cohort (*Sost;* ∼15-fold average decrease relative to the AngII No AAA cohort; *p *=* *5 × 10^−5^) had no differential expression in the infrarenal dataset. *Sost* encodes sclerostin,^60^ a secreted glycoprotein that can inhibit Wnt signaling, leading to decreased expression of osteopontin (*Spp1*), MMP-2, and MMP-9 (*61*). Our findings of significant downregulation of *Sost* accompanied by upregulated *Spp1* and *Mmp9* are in line with the study by Krishna *et al.* which demonstrated the potential of sclerostin in inhibiting dissecting AAA formation and atherosclerosis and provided evidence for epigenetic silencing of sclerostin in human AAAs.^61^

A secreted glycoprotein found in serum that is thought to be involved in differentiation of neutrophils (leucine-rich α2-glycoprotein, LRG)^62^ showed upregulated gene expression in both the AngII AAA and AngII No AAA cohorts relative to the Saline cohort (∼5.5- and 3-fold increase, respectively). Previous studies^63,64^ have proposed that LRG could be used as a circulating biomarker for inflammatory states. It would be of interest to validate whether LRG has higher circulating levels in animals that do develop a dissecting AAA.

## STUDY LIMITATIONS

Our study has several limitations that are important to note. While we did not visualize the thoracic aorta as part of the imaging protocol, AngII-induced dissections can also arise in this region.^65^ Therefore, it is possible that we overlooked focal dissections developing in the thoracic aorta of AngII-infused animals. However, we observed no thoracic aortic dissections after euthanasia and restricted our collection of aortic tissue to the abdominal aorta rather than the entire aortic tree, which would minimize the influence of any undetected pathological changes in the thoracic aorta. Furthermore, it is possible that minor microstructural defects led to moderate vessel expansion without false lumen formation in the suprarenal aorta.^20,66^ In this case, two-dimensional diameter measurements and one-dimensional vessel wall strain calculations would likely be inadequate to differentiate between a healthy and diseased suprarenal aorta. Indeed, as discussed above, this limitation underscores the importance of more sophisticated *in vivo* strain analysis in identifying diseased regions.^31^ Still, *in vivo* US imaging in the long- and short-axes of vessel wall pulsatility and blood flow dynamics, later confirmed by direct visualization after euthanasia, enabled us to correctly classify those animals with newly developed dissected aneurysmal lesions in the suprarenal aorta.

We chose to divide each aortic segment so that the tissue halves could be allocated for different types of experiments. The issue with this approach is that the proximal and distal regions of the suprarenal aorta have different properties. Our analysis of the disease-initiating effects is therefore more limited than it would be if we were to analyze both regions at the gene and tissue level. If, for example, a focal dissection arose adjacent to the right renal-mesenteric trifurcation, then the gene expression data could reflect the effects of dissection formation more so than the histology and immunohistochemistry results, which were analyzed on the proximal half of the aneurysmal lesion. A more advanced workflow that allows for RNA extraction from histology-prepped tissue, such as laser capture microdissection, could possibly circumvent this issue.

Finally, we designed this study to characterize how AngII differentially affects the suprarenal aorta of mice that have developed a dissecting AAA within the last 24 h as compared to those that did not develop one. What remains unclear to us is whether the effects we are measuring for the AngII AAA cohort are simply the response to injury or are also indicative of the disease initiation processes that may continue to be active in the midst of tissue healing and repair. Indeed, timing the collection of the tissue immediately before or after dissection formation would be very interesting for a future study. This, however, would be challenging as it would require an approach capable of predicting precisely when a dissection is likely to occur.

## SUMMARY AND FUTURE DIRECTIONS

We have quantitatively characterized the early pathology seen in AngII-infused *apoE*^-/-^ mice through a comparison with animals that did not develop dissections and saline-infused controls. We found that the aorta expanded and had reduced vessel wall strain regardless of whether a dissecting AAA formed in AngII-infused mice. Biological processes related to neutrophil and mononuclear cell inflammation, regulation of IL-1β signaling, ECM remodeling, and blood coagulation were all enriched in the AngII AAA cohort. Processes such as muscle contraction and wound healing were enriched in the AngII No AAA cohort relative to saline controls alongside intermediate gene expression of proinflammatory and matrix remodeling genes. Histology revealed the presence of focal elastin breakage, accumulation of fibrin and red blood cells, and relatively little adventitial collagen in early stage dissecting AAAs. Furthermore, we observed that neutrophils are co-distributed with IL-1β and MMP-9. This work advances our understanding of the early development of this murine model and has potential translational significance for improving diagnosis and therapy options of acute aortic syndrome in humans.

## METHODS

### Experimental design

The Purdue Animal Care and Use Committee approved all experiments performed on the animal protocol (#130200818). We implanted 20 male *apoE*^-/-^ C57BL/6J mice at 12 weeks of age with subcutaneous miniosmotic pumps (Alzet, DURECT Corp, Cupertino, CA) loaded with either AngII (n = 15) or saline (n = 5). AngII was loaded according to the animal body weight at a dose of 1000 ng/kg min for an infusion duration of 28 days. AngII-infused animals were monitored for dissecting AAA development by US for up to 10 days after pump implantation surgery. At the time of pump implantation (12 weeks of age), the AngII AAA cohort weighed 28.4 ± 1.7 g, the AngII No AAA cohort 27.5 ± 1.6 g, and the Saline cohort 28.8 ± 2.1 g. At the time of euthanizing (within 10 days after implantation), the AngII AAA cohort weighed 27.0 ± 1.4 g, the AngII No AAA cohort 26.7 ± 1.9 g, and the Saline cohort 29.5 ± 1.6 g. Individual weight fluctuations relative to the time of pump implantation did not vary by more than 10% over the study period.

### *In vivo* ultrasound imaging and blood pressure collection

Before implanting pumps (baseline), we collected full US imaging datasets and systemic pressures. Starting at day 3 post-implantation, we screened the AngII-infused animals daily for the appearance of dissecting AAAs (Table I and supplementary material, Fig. 1). We acquired *in vivo* US data of aortic size (B-mode), pulsatility (M-mode), and blood flow dynamics (Color Doppler) using a 50 MHz center frequency transducer (MS700) on the Vevo2100 system (FUJIFILM VisualSonics, Toronto, Canada). When screening an animal, if both short- and long-axis suprarenal aortic diameters were the same the previous day, we collected B-mode, M-mode, and Color Doppler scans and continued screening the following day. If the diameter was larger, we checked whether the aortic wall motion was substantially reduced (M-mode) and whether there was any disturbance in blood flow along the suprarenal aorta (Color Doppler). If a suprarenal aortic dissection was visible, we collected a full imaging dataset (2D and 3D B-mode, M-mode, Color Doppler, PW Doppler, and EKV) of the suprarenal aorta and euthanized the animal (supplementary material, Movie I). By day 10, if an aortic dissection was not observed, as would be evidenced by a substantial increase in the diameter alongside vessel stiffening and disturbance in blood flow, we collected a full imaging dataset and euthanized the animal (supplementary material, Movie I). For saline-infused mice, we collected imaging data at day 10 and then euthanized. US analysis (VEVO Lab, FUJIFILM VisualSonics) included EMD measurement, inner diameter measurement to calculate CCS, and 3D segmentation to measure the volume/length. We identified the location of maximum suprarenal aortic expansion seen in the short axis and measured the area bound by the outer aortic wall. From this value, we calculated EMD using the equation for the area of a circle. At the same location, we determined average peak systolic and average end diastolic inner diameters from M-mode data using a custom MATLAB script.^67^ With these values, we calculated Green-Lagrange circumferential cyclic strain, as described previously.^14^ With 3D B-mode scans, we segmented both the true lumen and the outer aortic wall. For the AngII AAA cohort, we segmented over the axial length of the dissecting AAA. Similarly for the AngII No AAA and Saline cohorts, we segmented 8 mm proximal to the right renal artery. We calculated volume/length values as the volume normalized to the segmentation length.

In addition to baseline assessment, we collected blood pressure measurements (CODA System, Kent Scientific, Torrington CT) from the tails of conscious animals between days 5 and 9. Each reported value is the average of systolic blood pressure measurements recorded from at least 11 acceptable runs with measured flow of more than 4 *μ*l/min.

### Animal euthanasia and tissue collection

We euthanized animals within 24 h of each animal-specific endpoint. We harvested the suprarenal aorta (from just distal to the diaphragm to just distal to the right renal artery) and the infrarenal aorta (from just distal to the left renal artery to the iliac bifurcation). Each aortic segment was divided via axial cuts into approximately equal halves and then frozen for histology and gene expression analysis (see below). For animals without dissecting AAAs, we grossed approximately 8 mm of both the suprarenal and infrarenal aorta.

### Total RNA extraction, dilution, and pooling

Total RNA from the distal half of each aortic segment was extracted with TRIzol reagent and treated with DNase I prior to elution from spin columns using the Zymo Directzol RNA Miniprep kit and the RCC-5 columns. One *μ*L of RNase inhibitor was added to each extracted sample. We verified sample quality by absorbance (Nanodrop, Thermo Scientific, Waltham, MA). We purified samples with suspected DNA or protein contamination using RNA Clean and Concentrator-5 kit (Zymo, Irvine, CA). For inclusion in cDNA library construction and real-time polymerase chain reaction (RT-PCR) assays, biological samples were required to have 260/230 and 260/280 absorbance ratios of greater than 1.7. We used fluorimetry (Qubit 2.0, Thermo Scientific) to determine sample concentrations.

Prior to cDNA library construction, we made 6 experimental pools, each consisting of either suprarenal (n = 3–7) or infrarenal (n = 2–5) aortic segments from one of the three cohorts. A given pool was comprised of samples with equal amounts of total RNA. Absorbance readings were taken after dilution and pooling, and clean-up was performed if necessary.

### cDNA library construction, purification, and quantification

We used the Ovation RNA-Seq System for mouse (NuGEN, San Carlos CA) designed for low input (10–100 ng) total RNA. Optional DNA fragmentation and concentration were not performed, but the libraries were depleted of ribosomal RNA using insert dependent adaptor cleavage (InDA-C) primers. We qualitatively assessed the amplified libraries using the Agilent Bioanalyzer 2100. If unreacted adapters or adapter dimers were present, we used Ampure beads to purify. Absolute size-adjusted concentrations for the libraries (8.2–87 nM) were determined using the KAPA Library Quantification Kit (KAPA Biosystems) and average library sizes from the electropherograms.

### RNA sequencing and bioinformatics

RNA sequencing (RNA-seq) was performed at the Purdue University Genomics Core Facility. Samples were equally pooled and clustered on an Illumina Rapid Chemistry flow cell. One and a half lanes were loaded, and samples were sequenced in single-read mode for 61 base reads on an Illumina HiSeq2500. The total number of reads per library was between 33.8 and 42.4 million.

We performed subsequent expression analysis on Galaxy (usegalaxy.org). RNA-seq reads were quality-filtered with FastQC, aligned with HISAT2, and assembled using the Cufflinks protocol.^68^ The data discussed in this publication have been deposited in NCBI's Gene Expression Omnibus and are accessible through GEO Series accession number GSE123874 (https://www.ncbi.nlm.nih.gov/geo/query/acc.cgi?acc=GSE123874). We first performed bioinformatics analysis for DEGs and GO terms using Advaita Bio's iPathwayGuide (http://www.advaitabio.com/ipathwayguide). This software analysis tool implements the “Impact Analysis” approach that takes into consideration the direction and type of all signals on a pathway, the position, role, and type of every gene, etc., as described by Draghici *et al.*^69^ We also used Qiagen Ingenuity Pathway Analysis (IPA) to generate networks and functional analyses (https://www.qiagenbioinformatics.com/products/ingenuity-pathway-analysis/) for the 6 datasets as well as for publicly available data published by Rush *et al.*^24^ (data accessible at NCBI GEO database, accession GSE12591). We utilized DAVID (Database for Annotation, Visualization and Integrated Discovery; https://david.ncifcrf.gov) to cluster genes by similar functional annotations collected from multiple sources, including GO terms, and to calculate an enrichment score for each functional annotation cluster.^70,71^

### Real-time PCR and relative gene expression analysis

Remaining total RNA was first reverse-transcribed to cDNA (Applied Biosystems High Capacity cDNA Reverse Transcription Kit, Waltham MA). We used ZEN double-quenched probes with fluorescein (6'-FAM) dye (Integrated DNA Technologies, Coralville, IA) to assay *mir223, Il1b, Eln, Col4a3, Mmp9*, and *Ppia*. We ran assays in duplicate using 0.5 ng of DNA template with the following cycling conditions: (1) 95 °C for 3 min; (2) 45 cycles of 95 °C for 15 s and 60 °C for 1 min (Applied Biosystems 7500 System). After validating constitutive expression of *Ppia* across experimental conditions, we selected it as a housekeeping gene for normalizing target gene expression levels. We performed relative quantitation of gene expression (n = 3–7 per cohort) by the ΔΔC_T_ method and used either the AngII No AAA or Saline cohort as a comparator group.

### Histology and immunohistochemistry

We embedded the proximal halves of suprarenal and infrarenal segments in optical cutting temperature (OCT) compound and froze the tissues over dry ice-cooled isopentane prior to storage at −80 °C. For each slide, 5 *μ*m-thick non-sequential cryosections (200 *μ*m apart) were collected starting from the distal end of each segment. Sections were fixed with methanol and stained using H&E, MOV, and MSB. Standard IHC was performed using the following primary antibodies: rabbit anti-mouse MMP-9 (ab38898; Abcam), goat anti-mouse IL-1β (AF401NA; R&D systems), rat anti-mouse neutrophil NIMP-R14 (ab2557; Abcam), and rat anti-mouse macrophage F4/80 (Cl:A3–1; Bio-Rad). Sections were developed using peroxidase substrate 3,3′diaminobenzidine (DAB), producing a brown stain in areas with immunoreactivity. Immunolabeling was quantified using marker-specific algorithms tuned by a pathologist to identify pixels with medium and strong DAB staining. We drew annotations of the vessel wall and excluded the lumen and periadventitial region from the analyzed areas. Positive pixel counts were normalized to the analyzed area on each section.

### Statistics

EMD and CCS values from each experimental cohort were compared by one-way ANOVA with Tukey multiple comparisons and correlated. For statistical analysis of ΔC_T_ values, we performed one-way ANOVA with Holm-Sidak multiple comparisons. Percent stained area values were compared between the AngII AAA and AngII No AAA cohorts by unpaired t-tests.

## SUPPLEMENTARY MATERIAL

See supplementary material for additional US image data and a molecule activity prediction map. Supplementary tables contain SBP measurements and lists of significant DEGs, GO terms, and unique DEGs found after comparison to a chronic AngII infusion study. A supplementary movie shows additional US image data.
